# Physical health in children with neurodevelopmental disorders

**DOI:** 10.1007/s10803-018-3697-4

**Published:** 2018-07-24

**Authors:** Setareh Alabaf, Christopher Gillberg, Sebastian Lundström, Paul Lichtenstein, Nóra Kerekes, Maria Råstam, Henrik Anckarsäter

**Affiliations:** 1 Gillberg Neuropsychiatry Centre, Institute of Neuroscience and Physiology, University of Gothenburg, Gothenburg, Sweden; 2 Center for Ethics, Law and Mental health (CELAM), Institute of Neuroscience and Physiology, University of Gothenburg, Gothenburg, Sweden; 3 Department of Medical Epidemiology and Biostatistics, Karolinska Institute, Stockholm, Sweden; 4 Department of Health Sciences, University West, Trollhättan, Sweden; 5 Department of Clinical Sciences Lund, Child and Adolescent Psychiatry, Lund University, Lund, Sweden

**Keywords:** Physical health, Neurodevelopmental disorders, Autism spectrum disorder (ASD), Attention-deficit/hyperactivity disorder (ADHD), Learning disorder (LD), Twins

## Abstract

With increasing numbers of children being diagnosed with neurodevelopmental disorders (NDDs) attention has been drawn to these children’s physical health. We aimed to identify the prevalence of defined physical problems (epilepsy, migraine, asthma, cancer, diabetes, psoriasis, lactose intolerance, celiac disease, diarrhea, constipation, daytime enuresis, encopresis) in a nationwide population of 9- and 12-year-old twins subdivided into those with and without indications of NDDs. Parents of 28,058 twins participated in a well-validated telephone interview regarding their children’s mental health and answered questions about their physical problems. The results indicate a high rate of physical problems in children with NDDs, particularly in those with indications of the presence of combinations of several NDDs.

**Electronic supplementary material:**

The online version of this article (10.1007/s10803-018-3697-4) contains supplementary material, which is available to authorized users.

## Introduction

Neurodevelopmental disorder (NDD) is a term used in the diagnostic and statistical manual of mental disorders, fifth edition (AmericanPsychiatricAssociation 2013) to describe a range of disorders with symptom onset during childhood. NDDs include—among other disorders—Autism spectrum disorder (ASD), which is characterized by impairment in social communication and inflexibility in interests, behavior and imagination (Lai et al. 2014), attention-deficit/hyperactivity disorder (ADHD), defined as problems with short attention span, impulsivity and/or hyperactivity (Pelham et al. 2005); and learning disorders (LD), most frequently displayed through symptoms of poor reading and/or writing ability (AmericanPsychiatricAssociation 2013).

The different NDDs share similar cognitive and behavioral processes, and frequently overlap with each other (and with other mental health disorders), which makes a dimensional approach to disorder classification useful (Anckarsater 2010). With this in mind, ESSENCE (early symptomatic syndromes eliciting neurodevelopmental clinical examinations) was introduced (Carlsson et al. 2013; Gillberg 2010) for the purpose of highlighting the significant amount of overlap across NDDs and the similarities of their symptoms mainly during young ages.

Recent research has shown a rise in the number of children clinically diagnosed with NDDs (Boyle et al. 2011) and that these children often have coexisting physical problems (Schieve et al. 2012). Until now, most studies have focused on physical problems co-occurring with either ASD or ADHD, such as the association between autism and epilepsy, which has been documented in many studies (Amiet et al. 2008; Jokiranta et al. 2014; Reilly et al. 2014). Besides epilepsy, the increased prevalence of gastrointestinal (GI) problems (Chaidez et al. 2014; McElhanon et al. 2014; Valicenti-McDermott et al. 2006), eating problems (Rastam et al. 2013), diarrhea and constipation (Chaidez et al. 2014), headache (Parisi et al. 2014) and asthma (Kotey et al. 2014) has been shown in children with ASD. A potential link between celiac disease and ASD has been investigated, but in most studies no such association has been found (Batista et al. 2012; Black et al. 2002; Lau et al. 2013). A case-control study reported elevated prevalence of specific immune-related comorbidities in ASD compared to controls; allergy, various autoimmune diseases and psoriasis (Zerbo et al. 2015). With regards to a link between type 1 diabetes mellitus (T1DM) and ASD, the results are diverse. One study from Finland could not find any increased occurrence of ASD among 5178 children with T1DM (Harjutsalo and Tuomilehto 2006), whereas a retrospective descriptive US study in young adults with ASD found a small but statistically significant increase in the prevalence of T1DM in ASD patients compared to general hospital patients (Kohane et al. 2012). A 35% increased risk of children with ASD to have asthma as compared to children in the general population was established in a large cross-sectional study in the United States (Kotey et al. 2014).

Some studies have shown increased prevalence of epilepsy (Cohen et al. 2013), migraine (Fasmer et al. 2011) and asthma (Fasmer et al. 2011) in patients diagnosed with ADHD, and vice versa increased prevalence of ADHD in clinical populations of children with somatic problems. For example, in children with asthma a significantly higher risk of developing ADHD during the school years has been detected compared to children without asthma (Chen et al. 2013). A recent Swedish study found that presence of ADHD made treatment of T1DM in teenagers a challenge (Lindblad et al. 2017) and a large epidemiological study found ADHD to be almost five times as common in children with enuresis as compared to children without (von Gontard et al. 2011). Using Norwegian national registries, a cross-sectional study found a significant association between ADHD and psoriasis, especially in females (Hegvik et al. 2018). A recent review on somatic comorbidity among adults with ADHD found that asthma is a well-documented comorbidity among adults with ADHD while the number of studies looking at the associations between ADHD and epilepsy, migraine, GI disorders and enuresis are still very few, ranging from one to three studies each (Instanes et al. 2018). A recent familial association case-control study of 117 children and adolescents with DSM-5 ADHD reported that a headache disorder diagnosis was common for both patients and healthy controls (59.0% vs. 37.8%), and migraine was found in 26.0% of ADHD cases and 9.9% of healthy controls (Kutuk et al. 2018). A positive association between the prescription of anti-migraine and ADHD medications and higher prevalence of migraine was found among adults with ADHD in Norway (Fasmer et al. 2012). One clinical study reported an overrepresentation of ADHD symptoms in patients with celiac disease (Niederhofer and Pittschieler 2006) and another study of patients with ADHD found an overrepresentation of celiac disease identified by the presence of celiac disease specific antibodies (Niederhofer 2011). In contrast, a large controlled study could not confirm the association between celiac disease and ADHD in children (Gungor et al. 2013).

There are few studies investigating physical health issues in children with LD or to what extent LD may exist in children with physical diseases (Schieve et al. 2012). One population-based study in Taiwan showed that the most common comorbid NDD among children with epilepsy was LD with a prevalence of 13% (Chiang and Cheng 2014). A Swedish study failed to show that children with T1DM had impaired academic ability (McCarthy et al. 2002).

In 2011, a population-based study was published investigating coexisting medical conditions in children with diagnoses of autism and/or ADHD (Schieve et al. 2012). This study found a moderate to high increase in the rate of most medical conditions examined in children with these NDDs. Phenotypical overlaps cannot be interpreted as causation or shared etiology per se, but a number of medical or genetic syndromes are known to cause physical problems and NDDs alike. Children with tuberous sclerosis have higher prevalence of ASD and learning disabilities (Mitchell et al. 2017) and studies have shown an association between early exposure of cranial radiation or chemotherapy and later development of neurocognitive problems (Anderson and Kunin-Batson 2009; Edelstein et al. 2011), but still the field of NDD in cancer patients is relatively unexplored. Even though comorbidity of NDDs is the rule rather than the exception (Gillberg 2010), no study has of yet specifically investigated the physical health of children with multiple overlapping NDDs, nor does a comprehensive overview of these associations exist.

In the present study, we used a Swedish nationwide general population cohort of twins to examine (a) the relationship between any “single” NDD and a variety of physical problems (epilepsy, migraine, asthma, cancer, diabetes, psoriasis, lactose intolerance, celiac disease, diarrhea, constipation, daytime enuresis, encopresis) and (b) if various constellations of NDDs are differently associated with these defined physical problems. The study provides a map of phenotypical associations between NDDs and physical problems, but does not assess underlying etiological factors, such as gestational age, birth weight or genetic mechanisms.

## Methods

### Subjects

Participants were included from the ongoing Child and Adolescent Twin Study in Sweden (CATSS) (Anckarsater et al. 2011) that aims to track all twins born in Sweden from 1992 and onwards. A telephone interview is conducted with the parents around the time of the children’s ninth birthday. The first three years of the CATSS interviews were conducted with parents of 12 year-old twins as well, to increase the number of birth cohorts included in the study. The entire CATSS-study has been described in detail in an overview article (Anckarsater et al. 2011). The present study comprises data on 28,058 children (all twins) born between the 31st of June 1992 through the 31st of December 2006, with a close to equal distribution of girls and boys (13,770 girls and 14,288 boys), and with over three times as many 9 year-olds as 12 year-olds (21,538 and 6520, respectively). Zygosity was determined by DNA sampling and use of questions on twin similarity (Hannelius et al. 2007). There were 7990 monozygotic (MZ), 9744 dizygotic same sex (DZss), 9722 dizygotic different sex (DZds) and 602 twins with unknown zygosity.

### Measures

#### A-TAC

The telephone interview in CATSS includes the Autism-Tics, ADHD and other Comorbidities (A-TAC), which is a fully structured interview designed to be used by laymen over the telephone. It consists of 96 questions, 17 of which correspond to the “ASD module”, 19 to the “ADHD module”, and 3 to the “LD module”. Each question has four response options: “no” (0), “yes, to some extent” (0.5), “yes” (1) or “I don’t know/I do not wish to answer” (missing). The A-TAC was developed based on the diagnostic criteria of DSM-IV, therefore its ASD module covers social interaction, language-communication, and flexibility-repetitive behavior problems in children, its ADHD module covers attention and activity/impulsiveness problems while the LD module measures learning difficulties in reading, writing and mathematical skills but does not exclude children with intellectual disability. The ASD, ADHD and LD modules of A-TAC have been extensively validated against DSM-IV criteria and ICD codes F84.0, F84.1, F84.5 and F84.9 for ASD, F90 for ADHD and F70-F79 for LD (Hansson et al. 2005; Larson et al. 2013; Marland et al. 2017). Assessments of the predictive validity of these A-TAC modules have shown Areas Under the Curve (AUC) of 0.88 for ASD, 0.90 for ADHD and 0.74 for LD (fair to excellent overall validity) (Hansson et al. 2005). In the present study, the high cut-off of each module was used to identify children with these NDDs, providing higher specificity and lower sensitivity. For ASD, the cut-off of ≥ 8.5 has a specificity of 0.91 and a sensitivity of 0.61, for ADHD, the cut-off of ≥ 12.5 has a specificity of 0.93 and a sensitivity of 0.56 and for LDs, the cut-off = 3 has a specificity of 0.93 and a sensitivity 0.41 (Larson et al. 2010). Children meeting one or more of these cut-offs constituted the “***NDD group***”, while the “***Comparison group***” consisted of twins who did not meet any of them. We excluded all children from the comparison group who fulfilled even the low cut-off criteria for ASD, ADHD or LD, with the purpose to provide a fully negative control group. Low cut-offs for ASD (≥ 4.5, specificity 0.88, sensitivity 0.96), ADHD (≥ 6.0, 0.81/0.98) and LD (≥ 1.0, 0.75/0.88) were characterized by high sensitivity but lower specificity (Larson et al. 2010). To be able to view the specific questions asked, the English version of A-TAC can be accessed by visiting the following web page: http://gillbergcentre.gu.se/digitalAssets/1566/1566533_a-tac_english.pdf.

#### Physical Problems

Physical problems were also assessed during the telephone interviews using questions requiring simple yes or no answers from the parents. Examples are: “Has he/she ever had any of the following diagnoses or disabilities”: Epilepsy? Migraine? Asthma? Cancer, tumor or leukemia? Diabetes? Psoriasis? Lactose intolerance? Celiac disease? Diarrhea? Constipation? In addition, two questions were used as proxies for daytime enuresis and for encopresis: “Has he/she during daytime wet himself/herself on several occasions after the age of five?” and Has he/she soiled himself/herself on several occasions after the age of four, except during gastroenteritis?

#### Study Groups

**Fig. 1:**
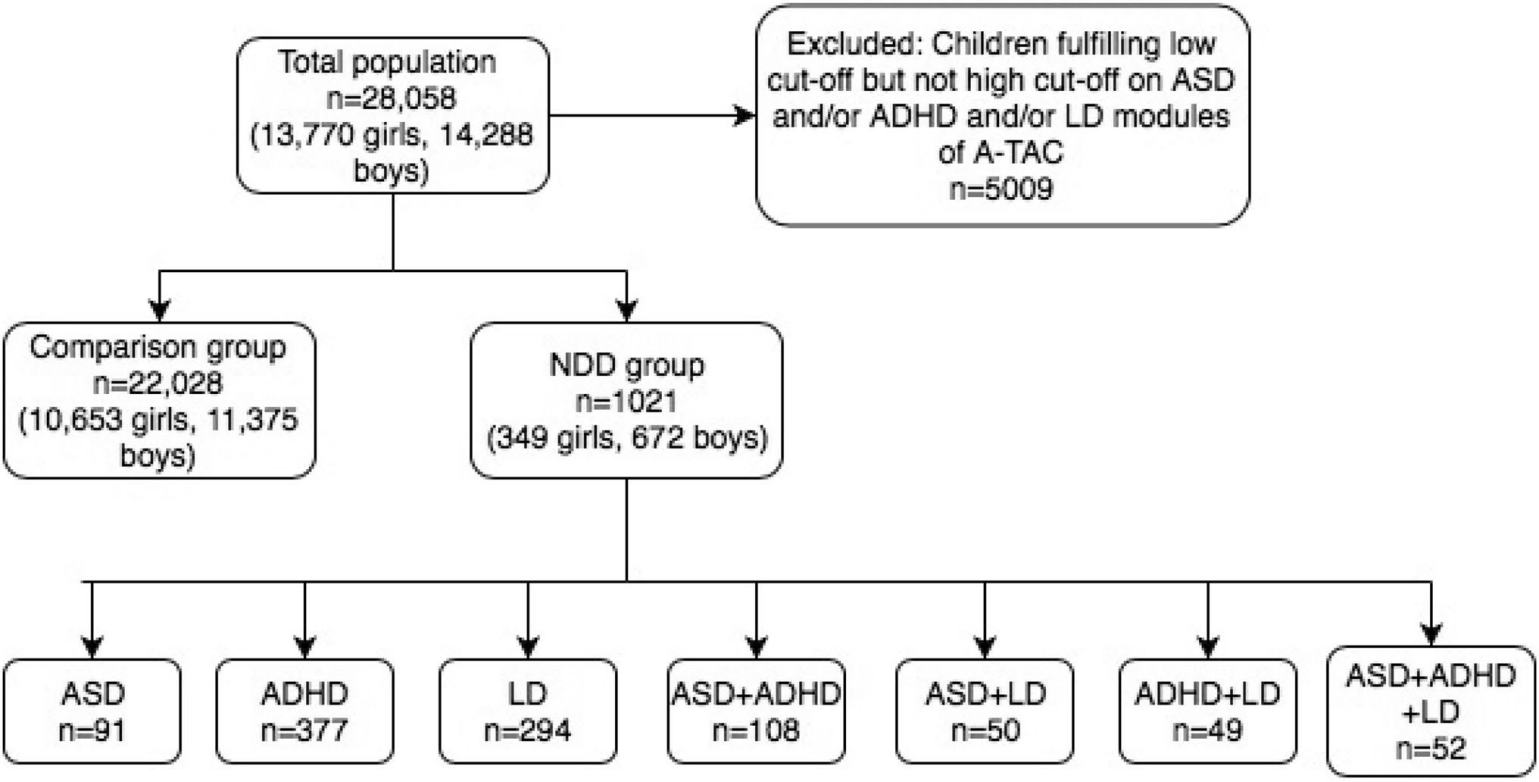
Flowchart of the study groups. In the present study, A-TAC high cut-offs were used as clinical proxies for ASD, ADHD and LD, which are included in the NDD group. The comparison group included only children who did not fulfill the low cut-off of any NDD. *A-TAC* autism-tics, ADHD and other comorbidities, *NDD* neurodevelopmental disorders, *ASD* autism spectrum disorder, *ADHD* attention-deficit/ hyperactivity disorder; *LD* learning disorder

(a) The NDD group included children whose scores reached the high cut-offs for ASD and/or ADHD and/or LD. In this group, the total number of children was 1021 [21% MZ twins, 672 (66%) boys and 349 (34%) girls]. Within this group, analyses of mutually exclusive separate diagnoses (ASD: 91 children, ADHD: 377 children and LD: 294 children) as well as by different constellations of several NDD diagnoses were conducted (ASD and ADHD n = 108, ASD and LD n = 50, ADHD and LD n = 49 and finally ASD and ADHD and LD n = 52). (b) The comparison group included children whose scores did not reach the low cut-offs in any of the ASD, ADHD or LD domains. This population consisted of 22,028 children (29% MZ twins, 10,653 (48%) girls and 11,375 (52%) boys). Categories are summarized in Fig. 1.

### Analyses

#### Statistical Analyses

Statistical analyses were performed using SPSS version 21. The Pearson Chi square or the Fisher´s exact tests were used to test for statistical significance between the prevalences of defined physical problems in groups and subgroups. P-values < 0.05 were considered statistically significant. Odds ratios (ORs), which were calculated from descriptive statistics, cross-tabulations, and by clicking risk and Cochran’s and Mantel-Haenzsel statistics in SPSS, were reported with 95% confidence interval. Figures were graphed using GraphPad Prism 7 software.

## Results

### Prevalence of Physical Problems in the General Population

**Fig. 2:**
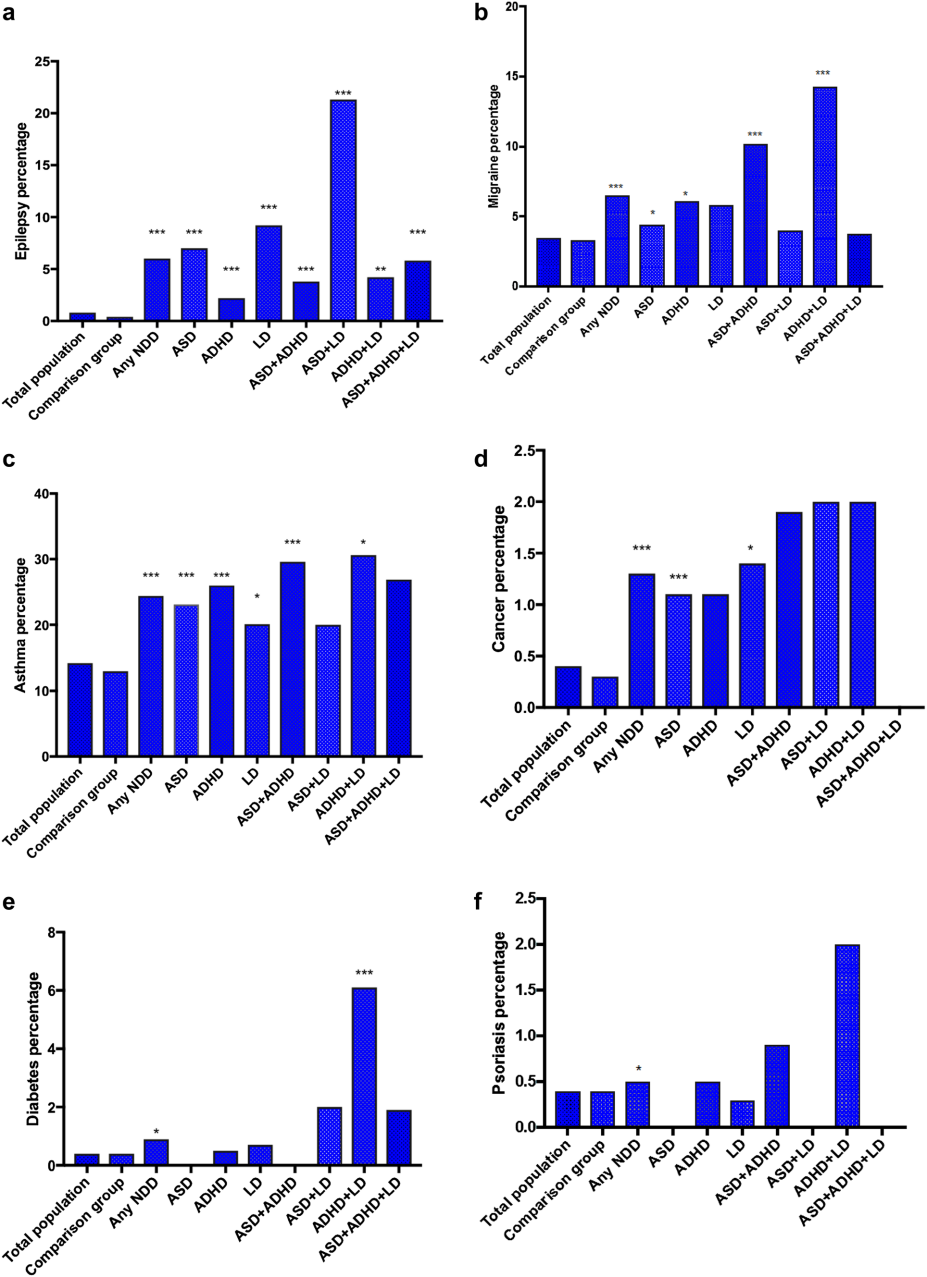
The figures show the prevalence of epilepsy (**a**), migraine (**b**), asthma (**c**), cancer (**d**), diabetes (**e**) and psoriasis (**f**) in our study groups and the statistical comparison to the comparison group consisting of twins who screened negative for neurodevelopmental disorders. Statistical significance is marked by stars, where p < 0.05 is represented by *, p < 0.01 by ** and p < 0.001 by ***. *NDDs* neurodevelopmental disorders, *ASD* autism spectrum disorder, *ADHD* attention-deficit/hyperactivity disorder and *LD* learning disorder

**Fig. 3:**
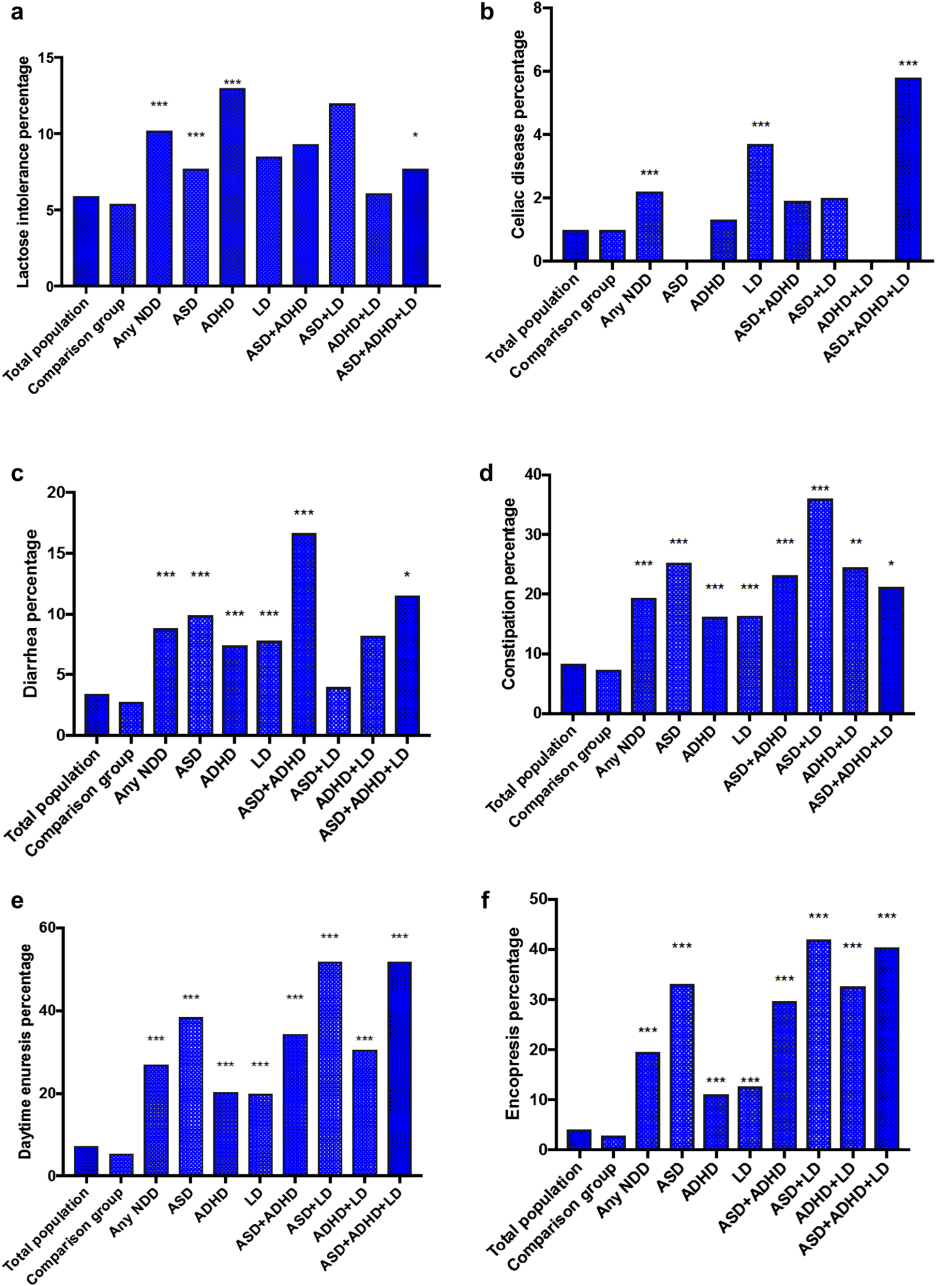
The figures show the prevalence of lactose intolerance (**a**), celiac disease (**b**), diarrhea (**c**), constipation (**d**), daytime enuresis (**e**) and encopresis (**f**) in our study groups and the statistical comparison to the comparison group consisting of twins who screened negative for neurodevelopmental disorders. Statistical significance is marked by stars, where p < 0.05 is represented by *, p < 0.01 by ** and p < 0.001 by ***. *NDDs* neurodevelopmental disorders, *ASD* autism spectrum disorder, *ADHD* attention-deficit/hyperactivity disorder and *LD* learning disorder

In this population of 9 or 12 year-old children, asthma had the highest prevalence (14.2%), followed by daytime enuresis (7.4%), encopresis (4.0%), and migraine (3.5%). Of the GI problems, the most prevalent was constipation (8.4%) and lactose intolerance (5.9%) (Figs. 2a–f, 3a–f). All physical problems were more common among children who screened positive for any NDD than among their screen negative peers.

### Prevalence of Physical Problems in the Groups with a Single NDD

Generally, children with NDDs had an elevated prevalence of most types of physical conditions, although not all of them reached statistical significance compared to the comparison group. The prevalence of physical problems in the defined groups is summarized in Figs. 2a–f and 3a–f, and detailed in the Supplementary material, Table 1.

Children who screened positive for a single NDD had significantly higher prevalences of epilepsy, diarrhea, constipation, daytime enuresis and encopresis (p < 0.001). For children with ASD, the OR of epilepsy and encopresis were the highest compared to comparison group, OR 9.9 for epilepsy and 12.1 for encopresis although prevalence of enuresis was the highest in the group (38.5%). Children who screened positive for LD only had significantly higher risk of epilepsy (OR 15) and cancer (OR 3.9), even though asthma, celiac disease, diarrhea, constipation, daytime enuresis and encopresis were also significantly more common than among controls. ADHD was associated with epilepsy, asthma, lactose intolerance, diarrhea, constipation, daytime enuresis and encopresis (p < 0.001) (supplementary material).

### Prevalence of Physical Problems in the Groups with Multiple NDDs

Children with a constellation of two or three NDDs more often reported physical problems than children with one NDD. However, there were no differences in the prevalence of physical problems between children with two versus all three NDDs. Some of the NDD constellations were associated with markedly increased prevalence of some specific physical problems. For example, children diagnosed with ASD and LD had an over 36 times increase in the prevalence of epilepsy (Fig. 2a, OR 36.2), six times increase in the prevalence of constipation (Fig. 3d, OR 6.2), almost 14 times increased prevalence of daytime enuresis (Fig. 3.d, OR 13.8), and over 17 times increased prevalence of encopresis (Fig. 3.e, OR 17.8). The coexistence of ASD and ADHD increased the odds for epilepsy (Fig. 2.a, OR 5.2) migraine (Fig. 2b, OR 3.2), asthma (Fig. 2c, OR 2.6), cancer (Fig. 2d, OR 5.2), diarrhea (Fig. 3c, OR 6.0), constipation (Fig. 3d, OR 3.4), daytime enuresis (Fig. 3e, OR 6.6), and encopresis (Fig. 3f, OR 10.4). In the group of children who were screen-positive for both ADHD and LD, the prevalence of diabetes was 15 times increased (Fig. 2e, OR 15.9) compared to the comparison group.

## Discussion

In this study, we found increased rates of physical problems in Swedish twins screening positive for NDDs as compared to controls. In particular, epilepsy, migraine, asthma, diarrhea, constipation, daytime enuresis and encopresis were overrepresented in children who were screen-positive for NDDs. These results are generally in line with previous studies finding increased rates of physical problems in children with NDDs (Amiet et al. 2008; Batista et al. 2012; Kohane et al. 2012; McKeown et al. 2013; Schieve et al. 2012). Our study advances the field showing that children with different constellations of NDDs more often had coexisting physical problems as compared to children with a single NDD. Moreover, our study indicates that some specific combinations carry higher risk of specific physical problems, which has been difficult to assess by the existing literature that has mostly examined the relationship between physical problems and specific NDDs.

Recently, the well-documented association between childhood epilepsy and ASD has been confirmed using the same twin study population as the present study (Gillberg et al. 2017). However, our results indicate that children with LD and especially children with both ASD and LD have the highest prevalence of epilepsy (OR 53), and that as many as 1/5 of them may be affected in childhood. The association between ASD and epilepsy may be partially mediated by the presence of LD or intellectual disability, also supported by previous research (Amiet et al. 2008).

Migraine and ASD include some symptomatic similarities, e.g. many children with ASD have sensory hypersensitivity (Sullivan et al. 2014), many migraine patients report symptoms such as photophobia during headache attacks (Hansen et al. 2015), and a recent study provides some preliminary evidence for a link between migraine, sensory hypersensitivity and anxiety in ASD (Sullivan et al. 2014). In the present study we found significant increase in the prevalence of migraine in children with ASD, ADHD and their combination as compared to the prevalence found in children without any NDD. It has been reported that some anti-migraine medications (which act through increasing serotonergic activity) can improve repetitive behavioral symptoms of autism (Hollander et al. 2000). Considering the overlap of migraine with ADHD, it has previously been suggested that migraine is not comorbid with ADHD in children, but with their hyperactive-impulsive behaviors (Arruda et al. 2010). In adult ADHD patients, the risk of migraine is increased by about 70% (Fasmer, Halmoy, Oedegaard, et al., 2011) which is a very similar increase to what was found here (OR 1.8). Also, the prescription pattern for anti-migraine medications and drugs for the treatment of ADHD in adults seems to indicate a link between migraine and ADHD (Fasmer et al. 2012).

Asthma was found to be very common among children with NDDs in our study, being most prevalent in children with both ADHD and LD where nearly one-third reported asthma. An American national survey on children below the age of 17 with a diagnosis of asthma, found, in line with our results, that asthma was independently associated with ADHD and LD (Blackman and Gurka 2007). Moreover, a study by Chen and co-workers indicated that asthma in early life among Taiwanese children predispose for ADHD later (Chen et al. 2013). The converse may also be true as studies indicate that ADHD is affected by regulation of the autonomic nervous system (Ward et al. 2015).

The association between cancer and central nervous system disorders has been explored in several population-based studies (Crawley et al. 2016; Driver et al. 2012; Ji et al. 2013). In our study, 1.3% of children with “any NDD” (as compared to 0.3% of children from the comparison group) had a parent-reported cancer history. Our result should be interpreted with caution as the number of individuals reporting cancer was small and that only living children were included. The link between cancer and NDD might speculatively be mediated through the various cancer treatments such as cranial radiation (Edelstein et al. 2011), even though we did not specifically monitor such treatments. A review by Anderson and co-workers indicated that even chemotherapy alone (without CNS irradiation) during childhood may have subtle long-term effects on attention and executive functioning later in life (Anderson and Kunin-Batson 2009). There may also be shared etiological factors, such as increased prevalence of mutations in PTEN (phosphatase and tensin homolog) that has been observed in ASD (McBride et al. 2010). PTEN is normally involved in regulating signaling pathways in cell proliferation and apoptosis (Worby and Dixon 2014) and mutations in PTEN could therefore theoretically increase the risk of cancer. In addition, it is also well known that many children with tuberous sclerosis (a proliferative genetic condition) develop ASD and/or have LD and a range of other developmental problems often conceptualized as “tuberous sclerosis-associated neuropsychiatric disorders” (de Vries et al. 2015). Similarly, in our study we found increased prevalence of cancer among children with ASD (1.1%) or LD (1.4%).

As previously mentioned, research has shown a link between maternal inflammatory conditions during pregnancy and offspring risk for NDDs (Ginsberg et al. 2017), both in mouse models (Mottahedin et al. 2017) and in clinical settings (Goines et al. 2011; Jiang et al. 2016). One study showed an association between maternal and paternal history of T1DM and ASD (Atladottir et al. 2009), while other studies indicate a possible link between autism and T1DM where 0.9% of children with T1DM in a tertiary hospital were also diagnosed with autism (Freeman et al. 2005). In our study, 0.4% of the comparison group (consisting of 22,028 children) screened positive for diabetes, and the prevalence of diabetes was significantly increased in children with NDD, especially ADHD. Studies have also shown significantly higher occurrence of autoimmune diseases (mainly hypothyroidism and rheumatic fever) in relatives of children with ASD, even higher than among family members to children with autoimmune disease (Sweeten et al. 2003). In a Swedish nationwide register-based cohort study in Sweden (Butwicka et al. 2017), children with biopsy-verified diagnosis of celiac disease had a 1.4-fold greater risk for mood disorders, anxiety disorders, eating disorders, behavioral disorders, ADHD, ASD, and intellectual disability compared to the general population. In contrast, siblings of celiac disease probands were at no increased risk for any of the investigated psychiatric disorders. These results are in alignment with our findings that suggest a possible link between autoimmune disorders such as celiac disease and NDDs.

Our findings included the strong positive association found between NDDs and GI problems, that is in line with a recent review on the association between ASD and gut microbiota (Li et al. 2017). Research indicates that the gut is physiologically related to the brain through the vagal nerve and the enteric nervous system, and that increases in the sympathetic tone, such as stress or anxiety, may decrease bowel movement in humans. Previous research has found a partial relationship between GI problems and higher level of anxiety (Mazefsky et al. 2014), sensory hyperactivity among children with NDDs (Mazurek et al. 2013), as well as increased heart rate variability and GI symptoms in children with ASD compared to controls (Ferguson et al. 2016). Whether these hypotheses can be extended to ADHD and/or LD remains to be better elucidated, but some results such as those presented here indicate that they probably can be.

The prevalence of daytime enuresis and encopresis in the present study was up to 50% in the NDD groups. The treatment of enuresis and encopresis in children with ASD, with or without LD, is very important (von Gontard et al. 2015). It has been shown that multimodal and individualized treatment in children with ADHD can treat the voiding symptoms (Kaye and Palmer 2010). One study indicated that children with ADHD and enuresis are less likely than children without ADHD to be brought to medical attention for their voiding problems (Shreeram et al. 2009). It is important to actively ask about incontinence because many children and/or their families may not easily report such problems. Many children with LD or autism also have impaired communicative abilities, which may further contribute to the high prevalence of enuresis and encopresis that we found in our study. We know that daytime enuresis and encopresis are common among school children with a prevalence of daytime enuresis among Swedish 10 year-olds estimated to be around 4%, while that for fecal incontinence/soiling is 7% in boys and 4% in girls (based on parental questionnaires), (Soderstrom et al. 2004). The prevalence of enuresis and encopresis reported in previous studies are highly variable due to different definitions and ascertainment (Peeters et al. 2013; Simonoff et al. 2008). Estimated prevalences of enuresis range from 5 to 43% in children with ADHD (Gor et al. 2012; Niemczyk et al. 2015; Robson et al. 1997; Shreeram et al. 2009) and from 11 to 55% among children with ASD (Gor et al. 2012; Simonoff et al. 2008; Taira et al. 1998). One study reported that almost one-third of pediatric patients with functional defecation disorder in a specialized outpatient setting had concomitant ASD symptoms (Peeters et al. 2013). Compared to prevalence estimates shown so far, our results are generally in line with earlier research and also indicate an increased risk for incontinence if the child has several NDDs.

## Clinical implications

It is known that comorbid physical conditions alter developmental trajectories (Hedvall et al. 2015). In agreement with the ESSENCE concept (Gillberg 2010, 2014) highlighting the significant amount of overlap across NDDs (Gillberg 2010), we recommend heightened awareness of the need for full medical/physical clinical examinations of children with NDDs.

We also recommend healthcare workers to specifically ask about GI problems and daytime enuresis as well as encopresis since these problems may be widespread in children with NDDs, causing medical and major social problems for the children and their families (Butler and Heron 2008). We also believe that it is of clinical importance to focus on all the different constellations of NDDs since our results indicate that these may be differently associated with physical problems.

## Conclusions

- Children with ASD, ADHD and/or LD have a high prevalence of physical comorbidities such as epilepsy, migraine, asthma, GI complications and incontinence.
- Our results indicate possible association between physical problems and constellations of NDDs as compared to single NDD diagnoses.

## Strengths and Limitations

Our study should be interpreted in the context of some strengths and limitations. The data are based on parental reports on psychiatric and physical problems and not on clinical assessment of the children, which is an obvious limitation. NDDs were assessed as proxy diagnoses using an instrument that was developed and validated for the purpose, the A-TAC (Hansson et al. 2005; Larson 2013; Larson et al. 2010, 2013). Regarding physical problems, simple questions have been used to detect the existence of these problems. This means that we did not have access to data that could disentangle and specify different subcategories of each problem type/diagnosis such as subtypes of epilepsy or of migraine. Previous validation studies have shown that simple questionnaires can be sufficient for screening purposes of at least some physical problems on population level (Gervil et al. 1998).

Our study population consisted of twins. Many twins are born preterm and there is an established association between prematurity and NDDs. However, although we did not correct for prematurity, we did not find a higher prevalence of NDDs (ASD 1.1%, ADHD, 2.1% and LD 1.6%) in our twin register similar to previous studies on twins (Croen et al. 2002; Hallmayer et al. 2002; Hultman et al. 2002). The strengths of our study are the very large study population and that it is systematically derived from the general population. The overlap between two conditions can be causative in either direction or due to shared susceptibility factors. It was beyond the scope of this article to investigate such confounding factors, e.g. pre-, peri- and postnatal factors in autism (Hisle-Gorman et al. 2018) or shared heritability.

## Electronic supplementary material

Below is the link to the electronic supplementary material.

Supplementary material 1 (DOCX 25 KB)
