# Correction to: Physical health in children with neurodevelopmental disorders

**DOI:** 10.1007/s10803-018-3758-8

**Published:** 2018-10-19

**Authors:** Setareh Alabaf, Christopher Gillberg, Sebastian Lundström, Paul Lichtenstein, Nóra Kerekes, Maria Råstam, Henrik Anckarsäter

**Affiliations:** 1 Gillberg Neuropsychiatry Centre, Institute of Neuroscience and Physiology, University of Gothenburg, Gothenburg, Sweden; 2 Center for Ethics, Law and Mental health (CELAM), Institute of Neuroscience and Physiology, University of Gothenburg, Gothenburg, Sweden; 3 Department of Medical Epidemiology and Biostatistics, Karolinska Institute, Stockholm, Sweden; 4 Department of Health Sciences, University West, Trollhättan, Sweden; 5 Department of Clinical Sciences Lund, Child and Adolescent Psychiatry, Lund University, Lund, Sweden

## Correction to: Journal of Autism and Developmental Disorders 10.1007/s10803-018-3697-4

The original version of this article unfortunately contained a mistake in Fig. 3 part labels, the label “d” was incorrectly labelled as “c” and the subsequent labels should be corrected as d, e, and f. The corrected Fig. 3 is given below.

**Fig. 3:**
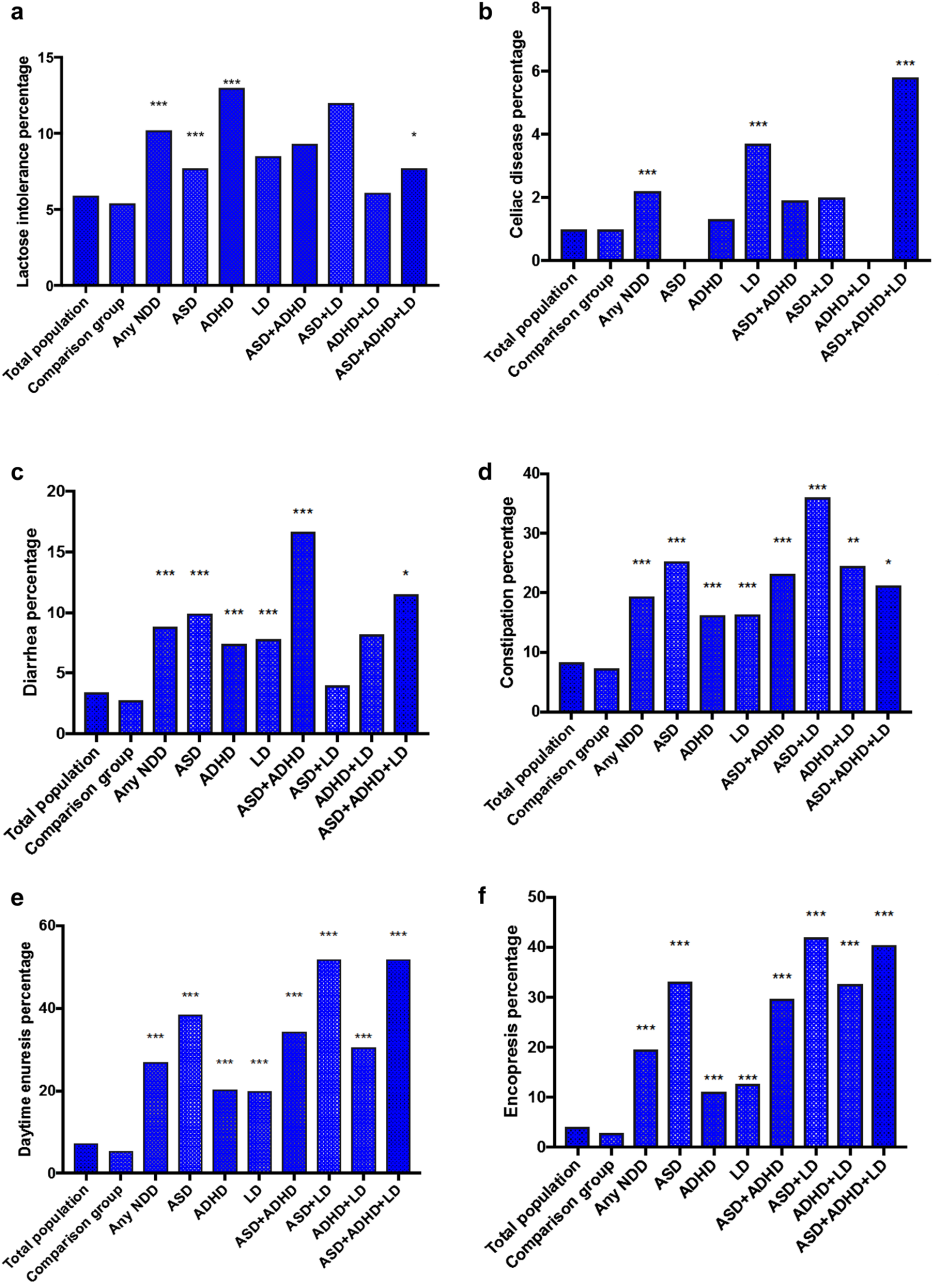
The figures show the prevalence of lactose intolerance (**a**), celiac disease (**b**), diarrhea (**c**), constipation (**d**), daytime enuresis (**e**) and encopresis (**f**) in our study groups and the statistical comparison to the comparison group consisting of twins who screened negative for neurodevelopmental disorders. Statistical significance is marked by stars, where p < 0.05 is represented by *, p < 0.01 by ** and p < 0.001 by ***. *NDDs* neurodevelopmental disorders, *ASD* autism spectrum disorder, *ADHD* attention-deficit/hyperactivity disorder and *LD* learning disorder

The original article has been corrected.

