# Supplementary material for: Physical health in children with neurodevelopmental disorders
Source: J Autism Dev Disord. 2018 Jul 24;49(1):83–95. doi: 10.1007/s10803-018-3697-4 (PMC6331488; doi:10.1007/s10803-018-3697-4)
Supplement: Supplementary file 1 — Supplementary material 1 (DOCX 25 KB) [file 10803_2018_3697_MOESM1_ESM.docx]

# Supplementary material

Appendix I

Table 1. Descriptive statistics of physical problems in defined subgroups in the study population

|  | *Total population*  *(n=28058)* | *Comparison group (n=22028)* | *Any NDD (n=1021)* | *ASD (n=91)* | *ADHD (n=377)* | *LD (n=294)* | *ASD+ADHD (n=108)* | *ASD+LD (n=50)* | *ADHD+LD (n=49)* | *ASD+ADHD+LD (n=52)* |
| --- | --- | --- | --- | --- | --- | --- | --- | --- | --- | --- |
| *Epilepsy* | **0.8%**  n=212 | **0.4%**  n=77 | **6.0%*****  n=60  OR=12.3  95%CI: 9.0-16.8 | **7.0%*****  n=6  OR=9.9  95%CI: 4.2-22.9 | **2.2%*****  n=8  OR=3.1  95%CI: 1.5-6.3 | **9.2%*****  n=27  OR=15.0  95%CI: 9.8-22.9 | **3.8%*****  n=4  OR=5.2  95%CI: 1.9-14.2 | **21.3%*****  n=10  OR=36.2  95%CI: 17.8-73.8 | **4.2%****  n=2  OR=5.7  95%CI: 1.4-23.5 | **5.8%*****  n=3  OR=7.9  95%CI: 2.4-25.6 |
| *Migraine* | **3.5%**  n=985 | **3.3%**  n=716 | **6.5%*****  n=66  OR=2.0  95%CI: 1.5-2.6 | **4.4%***  n=4  OR=1.3  95%CI: 0.5-3.6 | **6.1%***  n=23  OR=1.8  95%CI: 1.2-2.8 | **5.8%**  n=17  OR=1.7  p=0.1  95%CI: 1.0-2.8 | **10.2%*****  n=11  OR=3.2  95%CI: 1.7-6.0 | **4.0%**  n=2  OR=1.2  p=0.3  95%CI: 0.3-4.9 | **14.3%*****  n=7  OR=4.8  95%CI: 2.1-10.7 | **3.8%**  n=2  OR=1.1  p=0.3  95%CI: 0.3-4.7 |
| *Asthma* | **14.2%**  n=3989 | **13%**  n=2863 | **24.4%*****  n=249  OR=2.0  95%CI: 1.7-2.3 | **23.1%*****  n=21  OR=1.9  95%CI: 1.1-3.0 | **26%*****  n=98  OR=2.1  95%CI: 1.7-2.7 | **20.1%***  n=59  OR=1.5  95%CI: 1.1-2.0 | **29.6%*****  n=32  OR=2.6  95%CI: 1.7-3.9 | **20.0%**  n=10  OR=1.5  p=0.7  95%CI: 0.8-3.0 | **30.6%***  n=15  OR=2.6  95%CI: 1.4-3.9 | **26.9%**  n=14  OR=2.2  p=0.1  95%CI: 1.2-4.0 |
| *Cancer* | **0.4%**  n=103 | **0.3%**  n=69 | **1.3%*****  n=13  OR=3.9  95%CI: 2.2-7.0 | **1.1%*****  n=1  OR=3.1  95%CI: 0.4-21.2 | **1.1%**  n=4  OR=3.0  p=0.1  95%CI: 1.1-8.1 | **1.4%***  n=4  OR=3.9  95%CI: 1.4-10.6 | **1.9%**  n=2  OR=5.2  p=0.1  95%CI: 1.2-21.3 | **2.0%**  n=1  OR=5.6  p=0.3  95%CI: 0.8-40.7 | **2.0%**  n=1  OR=5.7  p=0.3  95%CI: 0.8-42.0 | **0%**  n=0  -  -  - |
| *Diabetes* | **0.4%**  n=117 | **0.4%**  n=91 | **0.9%***  n=9  OR=2.2  95%CI: 1.1-4.4 | **0%**  n=0  -  -  - | **0.5%**  n=2  OR=1.3  p=0.9  95%CI: 0.3-5.2 | **0.7%**  n=2  OR=1.6  p=0.9  95%CI: 0.4-6.7 | **0%**  n=0  -  -  - | **2.0%**  n=1  OR=4.9  p=0.4  95%CI: 0.7-35.8 | **6.1%*****  n=3  OR=15.9  95%CI: 4.9-51.9 | **1.9%**  n=1  OR=4.7  p=0.2  95%CI: 0.6-34.3 |
| *Psoriasis* | **0.4%**  n=116 | **0.4%**  n=87 | **0.5%***  n=5  OR=1.2  95%CI: 0.5-2.9 | **0%**  n=0  -  -  - | **0.5%**  n=2  OR=1.3  p=1.0  95%CI: 0.3-5.2 | **0.3%**  n=1  OR=0.8  p=1.0  95%CI: 0.1-5.9 | **0.9%**  n=1  OR=2.3  p=0.3  95%CI: 0.3-16.4 | **0%**  n=0  -  -  - | **2.0%**  n=1  OR=5.0  p=0.4  95%CI: 0.7-36.8 | **0%**  n=0  -  -  - |
| *Lactose intolerance* | **5.9%**  n=1641 | **5.4%**  n=1182 | **10.2%*****  n=104  OR=1.9  95%CI: 1.5-2.3 | **7.7%*****  n=7  OR=1.4  95%CI: 0.6-3.0 | **13%*****  n=49  OR=2.4  95%CI: 1.8-3.3 | **8.5%**  n=25  OR=1.5  p=0.2  95%CI: 1.0-2.3 | **9.3%**  n=10  OR=1.7  p=0.4  95%CI: 0.9-3.2 | **12.0%**  n=6  OR=2.2  p=0.3  95%CI: 1.0-5.2 | **6.1%**  n=3  OR=1.1  p=1.0  95%CI: 0.3-3.4 | **7.7%***  n=4  OR=1.4  95%CI: 0.5-3.9 |
| *Celiac disease* | **1.0%**  n=292 | **1.0%**  n=217 | **2.2%*****  n=22  OR=2.2  95%CI: 1.4-3.4 | **0%**  n=0  -  -  - | **1.3%**  n=5  OR=1.3  p=0.9  95%CI: 0.5-3.1 | **3.7%*****  n=11  OR=3.8  95%CI: 2.1-7.0 | **1.9%**  n=2  OR=1.8  p=0.6  95%CI: 0.4-7.4 | **2.0%**  n=1  OR=2.0  p=0.2  95%CI: 0.3-14.3 | **0%**  n=0  -  -  - | **5.8%*****  n=3  OR=6.2  95%CI: 1.9-20.1 |
| *Diarrhoea* | **3.4%**  n=940 | **2.8%**  n=622 | **8.8%*****  n=90  OR=3.0  95%CI: 2.4-3.8 | **9.9%*****  n=9  OR=3.3  95%CI: 1.7-6.6 | **7,4%*****  n=28  OR=2.4  95%CI: 1.6-3.5 | **7.8%*****  n=23  OR=2.5  95%CI: 1.6-3.9 | **16.7%*****  n=18  OR=6.0  95%CI: 3.6-10.0 | **4.0%**  n=2  OR=1.2  p=0.9  95%CI: 0.3-4.9 | **8.2%**  n=4  OR=2.6  p=0.3  95%CI: 0.9-7.1 | **11.5%***  n=6  OR=3.8  95%CI: 1.6-8.8 |
| *Constipation* | **8.4%**  n=2341 | **7.3%**  n=1600 | **19.4%*****  n=198  OR=2.8  95%CI: 2.4-3.3 | **25.3%*****  n=23  OR=3.8  95%CI: 2.4-6.2 | **16.2%*****  n=61  OR=2.2  95%CI: 1.7-2.9 | **16.3%*****  n=48  OR=2.2  95%CI: 1.6-3.0 | **23.1%*****  n=25  OR=3.4  95%CI: 2.1-5.3 | **36.0%*****  n=18  OR=6.2  95%CI: 3.4-11.1 | **24.5%****  n=12  OR=3.6  95%CI: 1.9-6.9 | **21.2%***  n=11  OR=2.9  95%CI: 1.5-5.7 |
| *Daytime enuresis* | **7.4%**  n=2065 | **5.4%**  n=1200 | **27.0%*****  n=276  OR=5.3  95%CI: 4.5-6.1 | **38.5%*****  n=35  OR=8.0  95%CI: 5.2-12.1 | **20.4%*****  n=77  OR=3.3  95%CI: 2.6-4.3 | **20%*****  n=59  OR=3.2  95%CI: 2.4-4.3 | **34.3%*****  n=37  OR=6.6  95%CI: 4.5-9.9 | **52%*****  n=26  OR=13.8  95%CI: 7.9-24.0 | **30.6%*****  n=15  OR=5.6  95%CI: 3.0-10.3 | **51.9%*****  n=27  OR=13.7  95%CI: 8.0-23.7 |
| *Encopresis* | **4.0%**  n=1118 | **2.7%**  n=609 | **19.5%*****  n=199  OR=7.0  95%CI: 5.9.-8.3 | **33%*****  n=30  OR=12.1  95%CI: 7.8-18.9 | **11.1%*****  n=42  OR=3.1  95%CI: 2.3-4.3 | **12.6%*****  n=37  OR=3.5  95%CI: 2.5-5.0 | **29.6%*****  n=32  OR=10.4  95%CI: 6.8-15.9 | **42%*****  n=21  OR=17.8  95%CI: 10.1-31.3 | **32.6%*****  n=16  OR=11.8  95%CI: 6.5-21.6 | **40.4%*****  n=21  OR=16.6  95%CI: 9.5-29.0 |

Table 1 summarizes the prevalence of targeted physical problems. Statistical significance is marked as p-value (* <0.05, ** <0.01, *** <0.001), odds ratio as OR with 95% confidence interval (95% CI). Abbreviations: Neurodevelopmental disorders (NDD), Autism Spectrum Disorder (ASD), Attention-Deficit/ Hyperactivity Disorder (ADHD) and Learning Disorder (LD)
